# Raising Concerns and Trainee Well-being: What Are the Issues?

**DOI:** 10.1192/bjo.2022.128

**Published:** 2022-06-20

**Authors:** Sabrina Hasnaoui, Vicki Ibbett, Sambavi Navaratnarajah, Rajendra Harsh, Shay-Anne Pantall, Ruth Scally

**Affiliations:** Birmingham and Solihull Mental Health NHS Foundation Trust, Birmingham, United Kingdom

## Abstract

**Aims:**

Raising concerns is an important part of being a doctor and part of the GMC ‘Good Medical Practice’ guidelines, however as a trainee it often comes with specific challenges. Historically trainees are often left feeling that their concerns are not taken seriously and there is no resolution to problems raised. Here we present the findings of a scoping exercise undertaken as part of a Quality Improvement project exploring these issues within a large mental health Trust.

**Methods:**

Trainees across all training grades from Foundation doctors to higher trainees were invited to engage in virtual focus groups specific to their training programme. Contributions were analysed anonymously using a thematic analysis approach by two independent coders. Quantitative data were also gathered using an online survey to capture trainees who had been unable to attend a focus group.

**Results:**

Key results included:

- A total of 6 focus groups were attended by more than 35 trainees, with high turnout particularly within Foundation trainees and CT1 doctors.
- Three key themes were identified from the qualitative data: difficulties with the process of raising concerns, fear of the consequences and challenging the culture of the organisation.
- It was noted that senior trainees felt more comfortable with the process of raising concerns compared with junior colleagues but were more apathetic about the impact of doing so.
- 12 trainees completed the online survey. Of these, 6 (50%) reported having had patient safety concerns and 7 (58%) had had concerns about their training.
- The most common reported barriers to raising concerns were the impact on working relationships (67%), lack of support (50%) and fear of repercussions on their training (50%). 42% of respondents were unsure of how to raise concerns.
- Trainee suggestions for change included improved information for trainees and trainers about the process for raising concerns, sharing of feedback about concerns raised more widely and regular opportunity to meet with key stakeholders.

**Conclusion:**

The majority of trainees had experienced concerns about either patient safety or training issues. It will be necessary to address the multiple barriers highlighted to enable trainees to feel more confident and able to raise concerns. Increasing awareness of escalation processes, improving the processes themselves and fostering a supportive environment which encourages and supports trainees to raise concerns will be important given the implications for patient safety and trainee well-being.

